# Draft Genome Sequence of Methicillin-Resistant *Staphylococcus aureus* Strain LC33 Isolated from Human Breast Milk

**DOI:** 10.1128/genomeA.00154-17

**Published:** 2017-04-13

**Authors:** Jéssica B. de Almeida, Suzi P. de Carvalho, Leandro M. de Freitas, Ana Marcia S. Guimarães, Naíla C. do Nascimento, Andrea P. dos Santos, Joanne B. Messick, Jorge Timenetsky, Lucas M. Marques

**Affiliations:** a State University of Santa Cruz (UESC), Campus Soane Nazaré de Andrade, Ilhéus, Brazil; b Multidisciplinary Institute of Health, Universidade Federal da Bahia, Vitória da Conquista, Brazil; c Department of Microbiology, Institute of Biomedical Science, University of São Paulo, São Paulo, Brazil; d Department of Comparative Pathobiology, Purdue University, West Lafayette, Indiana, USA

## Abstract

Here, we report the draft genome sequence of *Staphylococcus aureus* strain LC33, isolated from human breast milk in Brazil. This microorganism has been typed as ST1/t127/sccmecV. To our knowledge, this is the first draft genome sequence of a methicillin-resistant *S. aureus* strain isolated from human breast milk.

## GENOME ANNOUNCEMENT

Human breast milk is the source of nutrition for newborns in the first months of life (1). Over the past years, the isolation of *Staphylococcus aureus* from human milk has raised concern regarding newborn infection following milk ingestion (2, 3). *S. aureus* is the second leading cause of acquired infections in neonatal intensive care units worldwide (4). The clinical importance of *S. aureus* is attributed to its high virulence, its being able to cause superficial lesions and systemic infections, and its rapid development of drug resistance (5). Accordingly, methicillin-resistant *S. aureus* (MRSA) is an important pathogen that was first detected in hospitals (HA-MRSA), but has recently emerged in the community (CA-MRSA) (6, 7).

*S. aureus* strain LC33 was isolated from samples of human breast milk collected from a human milk bank in Vitória da Conquista, Bahia, Brazil. The strain was characterized by multilocus sequence typing as sequence type 1 (ST1), clonal complex 1 (CC1), as t127 by *spa* typing, and as carrying a staphylococcal chromosomal cassette *mec* element (SCC*mec*) type V by PCR. DNA was extracted using a PureLink Genomic DNA minikit (Life Technologies, Inc., Brazil), and the whole genome was sequenced from a paired-end library using the Illumina HiSeq 2500 platform (Illumina, Inc., San Diego, CA, USA) at the Purdue University Genomics Core Facility. Average reads of about 100 bases were assembled using ABySS version 1.2.7. After assembly resulting from 1,641× genome coverage, first-pass annotation was achieved using the NCBI Prokaryotic Genome Annotation Pipeline (PGAP).

A total of 39,477,532 reads were assembled into 53 contigs, resulting in a genome size of approximately 2,851 Mb with a G+C content of 32.7%. Annotation resulted in 2,860 protein-coding genes, 47 RNA-coding genes (39 tRNAs, four rRNAs, and four ncRNAs), and 84 pseudogenes. The resistome was analyzed using ResFinder version 2.1 (http://cbs.dtu.dk//services/ResFinder), and the following resistance genes were identified: *aph(3′)-III* (aminoglycoside resistance); *mecA* (β-lactam resistance); *blaZ* (β-lactam resistance) and *norA* (fluoroquinolone resistance). To our knowledge, the draft genome sequence presented here is the first for an *S. aureus* strain isolated from human breast milk. Further analyses of these data may contribute to a better understanding of the molecular mechanisms involved in antibiotic resistance, pathogenicity, and dissemination.

### Accession number(s).

The genome sequence of methicillin-resistant *S. aureus* strain LC33 has been deposited in GenBank under the accession no. MSFD00000000.
